# Standard error of the genetic correlation: how much data do we need to estimate a purebred-crossbred genetic correlation?

**DOI:** 10.1186/s12711-014-0079-z

**Published:** 2014-11-19

**Authors:** Piter Bijma, John WM Bastiaansen

**Affiliations:** Animal Breeding and Genomics Centre, Wageningen University, PO Box 338, 6700 AH Wageningen, The Netherlands

## Abstract

**Background:**

The additive genetic correlation (*r*_*g*_) is a key parameter in livestock genetic improvement. The standard error (SE) of an estimate of *r*_*g*_, ${\widehat{r}}_g$, depends on whether both traits are recorded on the same individual or on distinct individuals. The genetic correlation between traits recorded on distinct individuals is relevant as a measure of, e.g., genotype-by-environment interaction and for traits expressed in purebreds *vs*. crossbreds. In crossbreeding schemes, *r*_*g*_ between the purebred and crossbred trait is the key parameter that determines the need for crossbred information. This work presents a simple equation to predict the SE of ${\widehat{r}}_g$ between traits recorded on distinct individuals for nested full-half sib schemes with common-litter effects, using the purebred-crossbred genetic correlation as an example. The resulting expression allows *a priori* optimization of designs that aim at estimating *r*_*g*_. An R-script that implements the expression is included.

**Results:**

The SE of ${\widehat{r}}_g$ is determined by the true value of *r*_*g*_, the number of sire families (*N*), and the reliabilities of sire estimated breeding values (EBV):

$$ SE\left({\widehat{r}}_g\right)\kern0.36em \approx \kern0.36em \sqrt{\kern0.24em \frac{\kern0.24em \frac{1}{\rho_x^2{\rho}_y^2}\kern0.36em +\kern0.36em \left(1+\frac{0.5}{\rho_x^4}+\frac{0.5}{\rho_y^4}-\frac{2}{\rho_x^2}-\frac{2}{\rho_y^2}\right)\;{r}_g^2\kern0.36em +\kern0.36em {r}_g^4}{N-1}}, $$

where ${\rho}_x^2$ and ${\rho}_y^2$ are the reliabilities of the sire EBV for both traits. Results from stochastic simulation show that this equation is accurate since the average absolute error of the prediction across 320 alternative breeding schemes was 3.2%. Application to typical crossbreeding schemes shows that a large number of sire families is required, usually more than 100. Since $SE\left({\widehat{r}}_g\right)$ is a function of reliabilities of EBV, the result probably extends to other cases such as repeated records, but this was not validated by simulation.

**Conclusions:**

This work provides an accurate tool to determine *a priori* the amount of data required to estimate a genetic correlation between traits measured on distinct individuals, such as the purebred-crossbred genetic correlation.

**Electronic supplementary material:**

The online version of this article (doi:10.1186/s12711-014-0079-z) contains supplementary material, which is available to authorized users.

## Background

The additive genetic correlation is a key parameter in livestock genetic improvement and is defined as the correlation between breeding values of individuals for two distinct traits, say *x* and *y* [1],

$$ {r}_g=\frac{\sigma_{A_{xy}}}{\sigma_{A_x}{\sigma}_{A_y}}, $$

where ${\sigma}_{A_{xy}}$ denotes the covariance between the breeding values *A*_*x*_ and *A*_*y*_ of individuals, and ${\sigma}_{A_x}$ and ${\sigma}_{A_y}$ the additive genetic standard deviations. Estimation of *r*_*g*_ requires substantial amounts of data [2-4].

The standard error (SE) of the estimated genetic correlation depends on whether both traits are recorded on the same individual or on distinct individuals [2]. Examples of cases where both traits are recorded on distinct individuals are: (i) traits that are expressed in different environments, where *r*_*g*_ is a measure of the degree of genotype-by-environment interaction, (ii) traits that are expressed in males *vs*. females, such as sperm quality in bulls and milk yield in cows, (iii) traits that are expressed in live *vs.* dead animals, such as meat quality traits in fattening pigs and longevity of sows, and (iv) traits that are expressed in purebreds *vs*. crossbreds. This work considers the SE of the estimated genetic correlation between traits recorded on distinct individuals, with a focus on the purebred-crossbred genetic correlation.

In crossbreeding schemes, the ultimate goal is to improve the performance of the crossbred offspring of the pure breeding lines. With genotype-by-environment interaction and/or non-additive genetic effects, purebred performance is an imperfect predictor of crossbred performance. Thus, selection in crossbreeding schemes is ideally based on information recorded on crossbred relatives of the purebred selection candidates, or on a genomic reference population based on crossbred phenotypes [5-8]. However, phenotypic and pedigree data are not always routinely collected on crossbred individuals. The genetic correlation between the purebred and the crossbred trait (*r*_*pc*_) is the key parameter that determines the need for crossbred information. Hence, accurate estimation of *r*_*pc*_ [9,10] is required to decide on the strategy used for data recording.

A priori, the desired accuracy of an estimate of *r*_*pc*_ should be at least as high as for an ordinary genetic correlation. For example, when accuracies of purebred and crossbred EBV (estimated breeding values) are similar, the loss in response to selection due to relying on purebred rather than crossbred information is ~10% when *r*_*pc*_ is 0.9, but ~30% when *r*_*pc*_ is 0.7. To accurately identify such differences in *r*_*pc*_, the SE of the estimated correlation should not be greater than ~0.05.

Predicting the SE of estimates of the genetic correlation has been studied for many years [2-4,11]. In particular, Robertson [2] considered the SE of estimates of the genetic correlation between traits recorded on distinct individuals, such as *r*_*pc*_ [12], but only for cases with equal heritabilities and equal numbers of offspring for both traits. Moreover, the reports in [2-4,11] all considered half-sib designs, and did not allow for full-sib groups within half-sib families or for common-litter environmental effects.

In addition, existing prediction equations may not be readily accessible to applied breeders, because the full predictions are complex and expressed in terms of intra-class correlations, rather than heritabilities and common-litter variances. Simplified expressions do exist, but express the SE as being proportional to $\left(1-{r}_g^2\right)$ and are very inaccurate when *r*_*g*_ is close to 1, which may often be the case for a genotype-by-environment correlation or purebred-crossbred correlation [1,2,4]. With the computing power available today, stochastic simulations offer a solution, but they are still too time-consuming to use as a simple interactive tool. Thus, although the topic is somewhat outdated, for applied breeding it is still relevant to propose a simple prediction of the SE of estimates of genetic correlations.

Moreover, while the use of crossbred phenotypes has been limited in applied breeding programs because tracing pedigree relationships in a crossbred production environment is not trivial, it has recently regained attention because genomic relations are a solution for the cumbersome pedigree tracing process. The idea that building a training dataset with crossbred phenotypes will permit selection for crossbred performance is attractive and has revived interest in using crossbred phenotypes.

Here, we present a simple prediction equation for the SE of the estimated genetic correlation between traits recorded on distinct individuals, for nested full-half sib schemes with common-litter effects. This expression allows *a priori* optimization of designs that aim at estimating *r*_*g*_. To facilitate application, an R-script that implements the prediction is included in Additional file 1. Examples of sample sizes required to estimate *r*_*pc*_ are provided for a number of practical cases, but optimization of schemes is not considered extensively, since it can be easily done for specific cases using the R-script.

## Methods

### Analytical prediction of the SE of genetic correlation estimates

In the following, purebred and crossbred performance will be used as an example of two traits recorded on distinct individuals. Hence, subscript *p*, referring to purebred, will be used to denote one trait, and subscript *c*, referring to crossbred, to denote the other. However, the resulting expression will apply to the general case of a genetic correlation between traits recorded on distinct individuals.

Consider a population with phenotypic records on purebred and crossbred offspring of *N* sires. Each sire was mated to ${n}_{d_p}$ dams of its own line, each dam producing ${n}_{o_p}$ purebred offspring, and to ${n}_{d_c}$ dams of the other line, each dam producing ${n}_{o_c}$ crossbred offspring. Thus, a half-sib structure is present between purebreds and crossbreds, whereas full-sib families are nested within half-sib families within the purebreds and within the crossbreds.

For both purebreds and crossbreds, the trait model is given by:

$$ {P}_i={A}_i+{c}_i+{e}_i, $$

where *A*_*i*_ denotes the breeding value, *c*_*i*_ the common-litter effect, and *e*_*i*_ the environmental effect for trait *i* (purebred or crossbred). Hence, it is assumed implicitly that fixed effects can be estimated accurately. We do not model permanent environmental effects. Hence, a single observation per individual and a single litter per dam are assumed.

The estimate of the purebred-crossbred genetic correlation is given by:

$$ {\widehat{r}}_{pc}=\frac{{\widehat{\sigma}}_{A_{pc}}}{{\widehat{\sigma}}_{A_p}{\widehat{\sigma}}_{A_c}}, $$

where ${\widehat{\sigma}}_{A_{pc}}$ denotes the estimate of the purebred-crossbred genetic covariance, and ${\widehat{\sigma}}_{A_p}$ and ${\widehat{\sigma}}_{A_c}$ the estimates of genetic standard deviations. Throughout this article, symbols with hats (^) denote estimates, which are random variables, while symbols without hats denote the true parameters. The standard error of ${\widehat{r}}_{pc}$ was derived using a Taylor-series expansion of the expression for ${\widehat{r}}_{pc}$. The final result is presented in the main text, while derivations are in Additional file 2.

The resulting expression shows that the SE of the estimate of the purebred-crossbred genetic correlation is determined by the true value of *r*_*pc*_, the number of sire families, *N*, and the reliabilities of sire EBV,

1 $$ SE\left({\widehat{r}}_{pc}\right)\kern0.36em \approx \kern0.36em \sqrt{\kern0.24em \frac{\kern0.24em \frac{1}{\rho_p^2{\rho}_c^2}\kern0.36em +\kern0.36em \left(1+\frac{0.5}{\rho_p^4}+\frac{0.5}{\rho_c^4}-\frac{2}{\rho_p^2}-\frac{2}{\rho_c^2}\right)\;{r}_{pc}^2\kern0.36em +\kern0.36em {r}_{pc}^4}{N-1}}, $$

where ${\rho}_p^2$ is the reliability (*i.e.*, squared accuracy) of sire EBV for purebred performance, and ${\rho}_c^2$ the reliability of sire EBV for crossbred performance. Reliabilities of EBV are given by:

2 $$ {\rho}^2=\frac{{\scriptscriptstyle \raisebox{1ex}{$1$}\!\left/ \!\raisebox{-1ex}{$4$}\right.}{\sigma}_A^2}{\operatorname{var}\left(\overline{P}\right)}, $$

where $\overline{P}$ denotes the average phenotypic value of the progeny of a sire with a variance equal to:

3 $$ \operatorname{var}\left(\overline{P}\right)={\scriptscriptstyle \raisebox{1ex}{$1$}\!\left/ \!\raisebox{-1ex}{$4$}\right.}{\sigma}_A^2+\frac{{\scriptscriptstyle \raisebox{1ex}{$1$}\!\left/ \!\raisebox{-1ex}{$4$}\right.}{\sigma}_A^2+{\sigma}_c^2}{n_d}+\frac{{\scriptscriptstyle \raisebox{1ex}{$1$}\!\left/ \!\raisebox{-1ex}{$2$}\right.}{\sigma}_A^2+{\sigma}_e^2}{n_d{n}_o}, $$

where ${\sigma}_c^2$ denotes the common-litter variance and ${\sigma}_e^2$ the environmental variance. Thus, Equations 2 and 3 are used twice, once for purebreds and once for crossbreds. Instead of using Equations 2 and 3, empirical reliabilities from genetic evaluations, when available, can be substituted into Equation 1.

In the limiting case where the number of dams mated to a sire and the number of offspring per dam are large, so that ${\rho}_p^2={\rho}_c^2\to 1$, the expression reduces to:

4 $$ SE\left({\widehat{r}}_{pc}\right)\approx \frac{1-{r}_{pc}^2}{\sqrt{N-1}}, $$

which is the common expression for the SE of a simple correlation coefficient [13].

### Simulations

A limited number of scenarios was tested by estimation of *r*_*pc*_ in simulated data using ReML [14] and compared to results from analysis of the data using random-effects ANOVA with dam families nested within sire families [15] and to predictions from Equation 1. The simulated data consisted of sires with purebred and crossbred offspring. Crossbred offspring were from F1 females mated to a terminal sire line, i.e., three purebred lines were simulated, each with an *N*_*e*_ of 100. For each purebred line, 10 generations of pedigree were used. Purebred and crossbred phenotypes were simulated from multivariate normal distributions, for different values of ${h}_p^2$, ${h}_c^2$, and *r*_*pc*_. Genetic correlations were estimated with the ASReml software [16], using 200 replicates per scenario. Average $SE\left({\widehat{r}}_{pc}\right)$ as reported by ASReml and the standard deviation of ${\widehat{r}}_{pc}$ over the 200 replicates were calculated.

A large number of simulated scenarios was tested using ANOVA and compared to predictions from Equation 1. One thousand replicates of all factorial combinations of *N* = (50, 150), *n*_*dp*_ = 10, ${n}_{d_c}=\left(5,\kern0.24em 20\right)$, ${n}_{o_p}=8$, ${n}_{o_c}=\left(6,\;12\right)$, *r*_*pc*_ = (−0.8, − 0.4, 0, 0.4, 0.8), ${h}_p^2=\left(0.3,\;0.6\right)$, ${h}_c^2=\left(0.2,\kern0.24em 0.4\right)$, ${c}_p^2=0.05$ and ${c}_c^2=\left(0,\kern0.24em 0.1\right)$ were simulated (320 scenarios in total). Genetic parameters were estimated using ANOVA. Estimates of *r*_*pc*_ outside the boundaries of −1 and 1 were set to the nearest boundary.

## Results

### Accuracy of SE predictions

Concordance between the ReML and ANOVA estimates from the simulations was very high (Table 1). The SE from the ReML analyses were a little lower than the SE from the ANOVA estimates, which was expected because the ReML estimates used 10 generations of pedigree information, whereas the ANOVA estimates were based on a family structure of a single generation. Moreover, the SE of the ReML estimates were less precisely estimated because of the limited number of replicates (See footnote of Table 1). Because of computation time, more extensive evaluation of the accuracy of predictions from Equation 1 was based on the ANOVA estimates.

**Table 1:** **Comparison of predicted** $\mathbf{S}\mathbf{E}\left({\widehat{\mathbf{r}}}_{\mathbf{pc}}\right)$ **from Equation** 1 **to empirical estimates from ANOVA and to empirical and reported estimates from ASReml**

**Design**						$\mathbf{S}\mathbf{E}\left({\widehat{\mathbf{r}}}_{\mathbf{pc}}\right)$			
								^**5**^ **ASReml**	
*r*_***pc***_	${\mathbf{h}}_{\mathbf{p}}^{\mathbf{2}}$	${\mathbf{h}}_{\mathbf{c}}^{\mathbf{2}}$	***n***_***d***_	***n***_***o***_	***N***	**Equation** 1	^**1**^ **Anova**	^**2**^ **Reported**	^**3**^ **Empirical**
0.4	0.1	0.1	10	4	100	0.195	0.203	0.191	^4^0.250
0.8	0.5	0.5	10	4	100	0.065	0.066	0.061	0.060
0.0	0.3	0.3	20	4	100	0.120	0.123	0.118	0.127
0.4	0.3	0.3	20	4	100	0.104	0.104	0.103	0.105
0.0	0.1	0.1	10	4	200	0.145	0.146	0.143	0.146
−0.8	0.5	0.5	10	8	200	0.039	0.039	0.036	0.034
0.8	0.5	0.5	20	8	200	0.032	0.032	0.028	0.030

For ${\sigma}_c^2=0$; ^1^results are the SD among 1000 replicates of ${\widehat{r}}_{pc}$; ^2^results are the average of reported SE of 200 replicates; ^3^results are the SD among 200 replicates of ${\widehat{r}}_{pc}$; ^4^four replicates were fixed at the boundary of ${\widehat{r}}_{pc}$ 1; with these four estimates removed the SE equaled 0.216; ^5^Empirical SE from ASReml were based on 200 replicates only, and may therefore deviate from the true SE. With 200 replicates, the SE of the relative empirical SE, i.e. the SE of the ratio of the empirical SE over the true SE, equals $SE\;\left[\widehat{SE}\left({\widehat{r}}_{pc}\right)/SE\left({\widehat{r}}_{pc}\right)\right]$ = $1/\sqrt{2\times \left(200-1\right)}$ ≈0.05 [13]; thus a 5% error in predicted SE does not indicate a significant discrepancy between predictions and simulations, indicating that 200 replicates yield a limited accuracy of the empirical SE; when predicted $SE\left({\widehat{r}}_{pc}\right)$ is unbiased, the expected absolute relative error equals ≈ 3.5%, and a relative error >9.8% indicates a significant difference between empirical and predicted $SE\left({\widehat{r}}_{pc}\right)$ (*P* <0.05; two-sided, not accounting for multiple testing).

ANOVA estimates showed that the predicted SE from Equation 1 were accurate since the average absolute relative error across all schemes evaluated was equal to 3.2% (=100% × |predicted SE-simulated SE|/simulated SE; [see Additional file 3]). Sizeable errors occurred only for schemes for which estimates of genetic variances were near 0 in some replicates, which yielded extreme values for ${\widehat{r}}_{pc}$ (this occurred occasionally for schemes with *N* =50, ${h}_c^2=0.2$ and ${c}_c^2=0.1$). For those schemes, the maximum absolute relative error was 14%. These schemes are, however, of little practical relevance since their $SE\left({\widehat{r}}_{pc}\right)$ was around 0.25, which is far too high to be useful in practice.

### Required sample sizes

**Figure 1:**
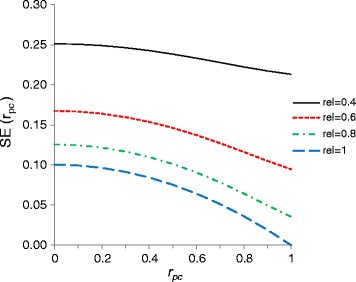
**Predictions of** $\mathbf{S}\mathbf{E}\left({\widehat{\mathbf{r}}}_{\mathbf{pc}}\right)$ **as a function of** ***r***_***pc***_ **, for different reliabilities of sire EBV (rel) that are assumed to be the same for the purebred and crossbred trait.** For *N* =100. The figure is symmetric in *r*_*pc*_, so the range for *r*_*pc*_ = −1 to 0 is omitted. $\mathrm{S}\mathrm{E}\left({\widehat{r}}_{pc}\right)$ was based on Equation 1.

Figure 1 shows predictions of $\mathrm{S}\mathrm{E}\left({\widehat{r}}_{pc}\right)$ based on Equation 1 as a function of *r*_*pc*_ for a sample size of 100 sires, and for different reliabilities of sire EBV. When sire EBV have high reliability, $\mathrm{S}\mathrm{E}\left({\widehat{r}}_{pc}\right)$ becomes considerably smaller when *r*_*pc*_ comes closer to 1. However, when sire EBV are inaccurate there is only a weak relationship between $\mathrm{S}\mathrm{E}\left({\widehat{r}}_{pc}\right)$ and *r*_*pc*_. Clearly, a sample of 100 half-sib families is too small, unless reliabilities of sire EBV are close to 1 and *r*_*pc*_ is greater than ~0.7.

**Figure 2:**
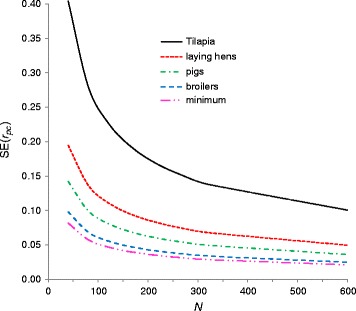
**Predictions of** $\mathbf{S}\mathbf{E}\left({\widehat{\mathbf{r}}}_{\mathbf{pc}}\right)$ **for typical breeding schemes as a function of the number of half-sib families (** ***N*** **).** $\mathrm{S}\mathrm{E}\left({\widehat{r}}_{pc}\right)$ were based on Equation 1 with the following input values: Harvest weight in aquaculture (tilapia): *n*_*d*_ = 2, *n*_*o*_ = 40, *h* ^2^ = 0.3, *c* ^2^ = 0.15, *r*_*pc*_ = 0.8. Egg number in laying hens: *n*_*d*_ = 7, ${n}_{o_p}=10$, ${n}_{o_c}=5$, *h* ^2^ = 0.2, *c* ^2^ = 0, *r*_*pc*_ = 0.6. Growth rate in pigs: *n*_*d*_ = 10, *n*_*o*_ = 10, *h* ^2^ = 0.3, *c* ^2^ = 0.10, *r*_*pc*_ = 0.7. Growth rate in broilers: *n*_*d*_ = 12, ${n}_{o_p}=70$, ${n}_{o_c}=10$, *h* ^2^ = 0.3, *c* ^2^ = 0.05, *r*_*pc*_ = 0.8. Minimum: Lowest possible $\mathrm{S}\mathrm{E}\left({\widehat{r}}_{pc}\right)$ for *r*_*pc*_ = 0.7 refers to a scheme with many dams per sire and many offspring per dam.

Figure 2 shows predictions of $\mathrm{S}\mathrm{E}\left({\widehat{r}}_{pc}\right)$ as a function of the number of half-sib families, for a range of schemes that may represent practical cases (personal communication Egiel Hanenberg, Gosse Veninga, Hooi Ling Khaw and Jeroen Visscher). Results from aquaculture breeding programs, such as for Tilapia, show that the commonly used strategy of mating a sire to only two dams, together with the presence of common full-sib family effects, causes very large standard errors, even when 600 half-sib families are used. On the contrary, the use of large numbers of dams per sire in broiler chicken breeding causes standard errors to approach their theoretical minimum (Equation 4).

## Discussion

The main objective of this work was to provide breeders with a simple tool to predict the SE of estimates of the genetic correlation between traits recorded on distinct individuals ($SE\left({\widehat{r}}_{pc}\right)$). The objective was not to address theoretical issues underlying the SE of genetic correlation estimates, which have been discussed extensively in the past [2-4,11]. Nevertheless, this work provides new insight on the impact of the reliability of sire EBV on $SE\left({\widehat{r}}_{pc}\right)$, which was not obvious from previous work. Equation 1 shows that $SE\left({\widehat{r}}_{pc}\right)$ depends on the reliabilities of sire EBV and the true value of *r*_*pc*_. Since Equation 1 is expressed in terms of reliabilities, it probably extends to other models for trait analysis, such as repeatability models, but this was not validated by simulation.

On the one hand, Equation 1 can be interpreted as a lower bound of $SE\left({\widehat{r}}_{pc}\right)$ because it assumes a balanced design and that the fixed effects are known, while actual estimation of *r*_*pc*_ always involves somewhat unbalanced data and estimation of fixed effects. However, on the other hand, Equation 1 assumes that *r*_*pc*_ is estimated from half-sib relationships only, whereas estimation of genetic parameters in livestock populations usually includes multiple generations of pedigree information, so that more distant relationships also contribute to the estimate, which reduces the SE.

We have considered a genetic correlation between traits measured on distinct individuals, of which the genetic correlation between purebred and crossbred performance, *r*_*pc*_, is an important example. When both traits are measured on the same individuals, additional complications arise due to covariances between the dam, common-litter and residual effects for the two traits. In such a case, derivation of $\mathrm{S}\mathrm{E}\left({\widehat{r}}_g\right)$ for a nested full-half sib scheme with common-litter effects is complicated, and this was not attempted here. When both traits are measured on the same individuals, stochastic simulation results (not shown) indicate that $\mathrm{S}\mathrm{E}\left({\widehat{r}}_g\right)$ is similar to the value given by Equation 1 when *r*_*g*_ = 0, but smaller than that value when the true correlation differs from 0. Hence, in most cases, the SE of a genetic correlation between traits measured on the same individuals is smaller than the value obtained from Equation 1.

Based on Robertson’s results [2], Falconer and Mackay [1] presented a simplified prediction of $\mathrm{S}\mathrm{E}\left({\widehat{r}}_g\right)$, taking the form $SE\left({\widehat{r}}_g\right)=\left(1-{r}_g^2\right)\;x$, where *x* is a function of the data structure and heritabilities. For *r*_*g*_ = ± 1, this expression yields $SE\left({\widehat{r}}_g\right)=0$, which is very inaccurate unless the reliabilities of sire EBV are close to 1 (Figure 1). For *r*_*g*_ → 1 and equal reliabilities of sire EBV for both traits, Equation 1 reduces to:

5 $$ SE\left({\widehat{r}}_g\Big|{r}_g=1\right)\approx \frac{\sqrt{2}}{\sqrt{N-1}}\kern0.24em \left(\frac{1}{\rho^2}-1\right), $$

which does not approach 0 unless reliabilities approach 1 (see values for *r*_*pc*_ = 1 in Figure 1).

## Conclusions

This paper presents a simple and accurate prediction of the standard error of estimates of the genetic correlation between traits recorded on distinct individuals, for nested full-half sibs schemes with common-litter effects. This allows breeders to decide on the required sample size to estimate this correlation, *e.g.*, to support decisions on the collection of crossbred information. Results show that more than 100 half sib families are required in most cases.

## Additional files

Additional file 1
**R-code for SE(rpc).** This file contains an R-script that implements Equation 1 for a range of input values of genetic parameters and breeding designs. Additional file 2
**Derivation of Equation** 1 **.** This file contains the derivation of Equation 1. Additional file 3
**Numerical validation of Equation** 1 **.** This file shows a comparison of predicted and empirical SE for a range of alternative schemes.
