# Supplementary material for: Standard error of the genetic correlation: how much data do we need to estimate a purebred-crossbred genetic correlation?
Source: Genet Sel Evol. 2014 Nov 19;46(1):79. doi: 10.1186/s12711-014-0079-z (PMC4236448; doi:10.1186/s12711-014-0079-z)
Supplement: Additional file 2 — Derivation of Equation 1 . This file contains the derivation of Equation 1. [file 12711_2014_79_MOESM2_ESM.pdf]

Additional file 2: Derivation of Equation 1

Part A

Consider $y = ab^{-0.5}c^{-0.5}$, where y represents $\hat{r}_{pc}$, a represents $\hat{\sigma}_{A_{pc}}$, being the purebred-crossbred genetic covariance, b represents $\hat{\sigma}^2_{A_p}$, and c represents $\hat{\sigma}^2_{A_c}$. A first-order tri-variate Taylor-series approximation at (μ_a, μ_b, μ_c), μ denoting the mean, yields

$$y \approx \mu_y + \mu_b^{-0.5}\mu_c^{-0.5}(a-\mu_a) - 0.5\mu_a\mu_b^{-1.5}\mu_c^{-0.5}(b-\mu_b) - 0.5\mu_a\mu_b^{-0.5}\mu_c^{-1.5}(c-\mu_c).$$

Taking the variance yields

$$\begin{aligned}\mathrm{var}(y) \approx\ & \mu_b^{-1}\mu_c^{-1}\sigma_a^2 \\ & -\mu_a\mu_b^{-2}\mu_c^{-1}\sigma_{ab} + \tfrac{1}{4}\mu_a^2\mu_b^{-3}\mu_c^{-1}\sigma_b^2 \\ & -\mu_a\mu_b^{-1}\mu_c^{-2}\sigma_{ac} + \tfrac{1}{2}\mu_a^2\mu_b^{-2}\mu_c^{-2}\sigma_{bc} + \tfrac{1}{4}\mu_a^2\mu_b^{-1}\mu_c^{-3}\sigma_c^2.\end{aligned}$$

Hence,

$$\begin{aligned}\mathrm{var}(\hat{r}_{pc}) \approx\ & \frac{\mathrm{var}(\hat{\sigma}_{A_{pc}})}{\sigma^2_{A_p}\sigma^2_{A_c}} \\ & -\frac{\sigma_{A_{pc}}}{\sigma^4_{A_p}\sigma^2_{A_c}}\mathrm{cov}(\hat{\sigma}_{A_{pc}},\hat{\sigma}^2_{A_p}) + \frac{\sigma^2_{A_{pc}}}{4\sigma^6_{A_p}\sigma^2_{A_c}}\mathrm{var}(\hat{\sigma}^2_{A_p}) \qquad \text{(A1)} \\ & -\frac{\sigma_{A_{pc}}}{\sigma^2_{A_p}\sigma^4_{A_c}}\mathrm{cov}(\hat{\sigma}_{A_{pc}},\hat{\sigma}^2_{A_c}) + \frac{\sigma^2_{A_{pc}}}{2\sigma^4_{A_p}\sigma^4_{A_c}}\mathrm{cov}(\hat{\sigma}^2_{A_p},\hat{\sigma}^2_{A_c}) + \frac{\sigma^2_{A_{pc}}}{4\sigma^2_{A_p}\sigma^6_{A_c}}\mathrm{var}(\hat{\sigma}^2_{A_c}).\end{aligned}$$

Calculating Equation A1 requires six elements: $\mathrm{var}(\hat{\sigma}_{A_{pc}})$, $\mathrm{var}(\hat{\sigma}^2_{A_p})$, $\mathrm{var}(\hat{\sigma}^2_{A_c})$, $\mathrm{cov}(\hat{\sigma}_{A_{pc}},\hat{\sigma}^2_{A_p})$, $\mathrm{cov}(\hat{\sigma}_{A_{pc}},\hat{\sigma}^2_{A_c})$, and $\mathrm{cov}(\hat{\sigma}^2_{A_p},\hat{\sigma}^2_{A_c})$.

var($\hat{\sigma}_{A_{pc}}$): Expressing trait values as deviations from their mean, $\hat{\sigma}_{A_{pc}} = \frac{4}{N-1}\sum_{i=1}^{N}\bar{P}_{i_p}\bar{P}_{i_c}$, where $\bar{P}_{i_p}$ denotes the average phenotype of the purebred offspring of the ith sire, and $\bar{P}_{i_c}$ the corresponding value for crossbreds. With unrelated sires, elements in the summation are independent, so that,

$$\mathrm{var}(\hat{\sigma}_{A_{pc}}) = \frac{16}{N-1}\mathrm{var}(\bar{P}_p\bar{P}_c),$$

in which $\bar{P}$ refers to the offspring of a single sire. Using $\mathrm{var}(xy) = \sigma_x^2\sigma_y^2 + \sigma_{xy}^2$ (Equation B1), and substituting $x = \bar{P}_p$, $y = \bar{P}_c$, and $\sigma_{xy}^2 = \mathrm{cov}^2(\bar{P}_p, \bar{P}_c) = \tfrac{1}{16}\sigma_{A_{pc}}^2$ yields

$$\mathrm{var}(\hat{\sigma}_{A_{pc}}) = \frac{16\,\mathrm{var}(\bar{P}_p)\,\mathrm{var}(\bar{P}_c) + \sigma_{A_{pc}}^2}{N-1}, \tag{A2}$$

in which $\mathrm{var}(\bar{P}_p)$ and $\mathrm{var}(\bar{P}_c)$ are the variances of the purebred and crossbred progeny averages of a sire, respectively. Variances of progeny averages are given by

$$\mathrm{var}(\bar{P}) = \tfrac{1}{4}\sigma_A^2 + \frac{\tfrac{1}{4}\sigma_A^2 + \sigma_c^2}{n_d} + \frac{\tfrac{1}{2}\sigma_A^2 + \sigma_e^2}{n_d n_o}, \tag{A3}$$

where σ_c^2 denotes the common-litter variance, and σ_e^2 the residual variance. Thus, Equation A3 is used twice; once for $\mathrm{var}(\bar{P}_p)$ using values for purebreds, and once for $\mathrm{var}(\bar{P}_c)$ using values for crossbreds.

$\mathbf{var}(\hat{\sigma}_{A_p}^2)$ and $\mathbf{var}(\hat{\sigma}_{A_c}^2)$: With two-way Anova, where dams are nested within sires, expected mean-squares are

$$EMS_{sires} = n_d n_o \sigma_{sire}^2 + n_o \sigma_{dam}^2 + \sigma_e^2,$$

$$EMS_{dams} = n_o \sigma_{dam}^2 + \sigma_e^2.$$

Hence,

$$\hat{\sigma}_{sire}^2 = \frac{MS_{sires} - MS_{dams}}{n_d n_o}.$$

Using $\mathrm{var}(MS) = 2EMS^2/df$ and $\mathrm{cov}(MS_{sire}, MS_{dam}) = 0$ [16], it follows that

$$\mathrm{var}(\hat{\sigma}_A^2) = \mathrm{var}(4\hat{\sigma}_{sire}^2) = 32\frac{EMS_{sire}^2/df_{sire} + EMS_{dam}^2/df_{dam}}{n_d^2 n_o^2}, \tag{A4}$$

with expected mean squares and degrees of freedom given by

$$EMS_{sires} = n_d n_o \sigma_{sire}^2 + n_o \sigma_{dam}^2 + \sigma_{error}^2,$$

$$EMS_{dams} = n_o \sigma_{dam}^2 + \sigma_{error}^2,$$

$$df_{sires} = N - 1,$$

$$df_{dams} = N(n_d - 1),$$

where

$$\sigma^2_{sire} = {}^1\!/_4\sigma^2_A ,$$

$$\sigma^2_{dam} = {}^1\!/_4\sigma^2_A + \sigma^2_c ,$$

$$\sigma^2_{error} = {}^1\!/_2\sigma^2_A + \sigma^2_e .$$

Thus, Equation A4 is used twice; once for $\mathrm{var}(\hat{\sigma}^2_{A_p})$ using values for purebreds, and once for $\mathrm{var}(\hat{\sigma}^2_{A_c})$ using values for crossbreds.

$\mathbf{cov}(\hat{\sigma}_{A_{pc}}, \hat{\sigma}^2_{A_p})$ and $\mathbf{cov}(\hat{\sigma}_{A_{pc}}, \hat{\sigma}^2_{A_c})$: To simplify the derivation, we introduce an effective number of sires for both purebred and crossbred performance, denoted $N_{eff,p}$ and $N_{eff,c}$. The effective number of sires is the number of sires with an infinitely large progeny group that would yield the same SE of the estimated additive genetic variance as the actual number of sires,

$$N_{eff} = \frac{2\sigma^4_A}{\mathrm{var}(\hat{\sigma}^2_A)} , \tag{A5}$$

where $\mathrm{var}(\hat{\sigma}^2_A)$ is taken from Equation A4. Thus Equation A5 is used twice, once for $N_{eff,p}$, and once for $N_{eff,c}$. When using this effective number, we can treat the estimated genetic variances as simple variances,

$$\hat{\sigma}^2_A \approx \frac{4}{N_{eff}} \sum_{N_{eff}} \overline{P}^2 \quad ,$$

where $\overline{P}$ denotes the progeny average of a sire, and trait values are expressed relative to their mean (*i.e.*, $\overline{\overline{P}} = 0$). Since the estimated purebred-crossbred genetic covariance arises only from the sire-terms, *i.e.*, does not involve MS_{dams} (see derivation of $\mathrm{cov}(\hat{\sigma}^2_{A_p}, \hat{\sigma}^2_{A_c})$ below), $\hat{\sigma}_{A_{pc}}$ depends on $N-1$ rather than N_{eff},

$$\hat{\sigma}_{A_{pc}} = \frac{4}{N-1} \sum_N \overline{P}_p \overline{P}_c .$$

Thus

$$\mathrm{cov}(\hat{\sigma}_{A_{pc}}, \hat{\sigma}^2_{A_p}) \approx \frac{16}{N_{eff,p}(N-1)} \mathrm{cov}\left[\sum_{N_{eff,p}} \overline{P}^2_p , \sum_N \overline{P}_p \overline{P}_c \right].$$

Since terms of different sires are independent, this can be approximated by

$$\text{cov}(\hat{\sigma}_{A_{pc}},\hat{\sigma}^2_{A_p}) \approx \frac{16\text{cov}(\overline{P}_p^2,\overline{P}_p\overline{P}_c)}{\sqrt{N_{eff,p}(N-1)}},$$

where the covariance term now refers to a single sire. From Equation B3, it follows that $\text{cov}(\overline{P}_p^2,\overline{P}_p\overline{P}_c) \approx \frac{1}{8}\sigma_{A_{pc}}\sigma^2_{A_p}$. This uses $\text{var}(\overline{P}_p) = \frac{1}{4}\sigma^2_{A_p}$, which is appropriate because of the division by $N_{eff,p}$, rather than $N-1$. Substituting this result into the expression for $\text{cov}(\hat{\sigma}_{A_{pc}},\hat{\sigma}^2_{A_p})$ yields

$$\text{cov}(\hat{\sigma}_{A_{pc}},\hat{\sigma}^2_{A_p}) = \frac{2\sigma_{A_{pc}}\sigma^2_{A_p}}{\sqrt{N_{eff,p}(N-1)}}. \quad \text{(A6a)}$$

Analogously,

$$\text{cov}(\hat{\sigma}_{A_{pc}},\hat{\sigma}^2_{A_c}) = \frac{2\sigma_{A_{pc}}\sigma^2_{A_c}}{\sqrt{N_{eff,c}(N-1)}}. \quad \text{(A6b)}$$

$\mathbf{cov(\hat{\sigma}^2_{A_p},\hat{\sigma}^2_{A_c})}$**:** Using $\hat{\sigma}^2_A = 4\frac{MS_{sires}-MS_{dams}}{n_d n_o}$, it follows that

$$\text{cov}(\hat{\sigma}^2_{A_p},\hat{\sigma}^2_{A_c}) = 16\text{cov}\left(\frac{MS_{p,sires}-MS_{p,dams}}{n_{d_p}n_{o_p}},\frac{MS_{c,sires}-MS_{c,dams}}{n_{d_c}n_{o_c}}\right).$$

Using $\text{cov}(MS_{sires},MS_{dams}) = 0$ [16], and $\text{cov}(MS_{p,dams},MS_{c,dams}) = 0$ since purebreds and crossbreds descend from different dams, it follows that

$$\text{cov}(\hat{\sigma}^2_{A_p},\hat{\sigma}^2_{A_c}) = 16\text{cov}\left(\frac{MS_{p,sires}}{n_{d_p}n_{o_p}},\frac{MS_{c,sires}}{n_{d_c}n_{o_c}}\right).$$

Next, from $MS_{sires} = SS_{sires}/(N-1)$ and $SS_{sires} = n_d n_o\sum_{i=1}^{N}(\overline{P}_{sire}-\overline{\overline{P}})^2$, it follows that

$$\text{cov}(\hat{\sigma}^2_{A_p},\hat{\sigma}^2_{A_c}) = \frac{16}{(N-1)^2}\text{cov}\left(\sum_{i=1}^{N}(\overline{P}_{p,sire}-\overline{\overline{P}}_p)^2,\sum_{i=1}^{N}(\overline{P}_{c,sire}-\overline{\overline{P}}_c)^2\right).$$

Since different sires are assumed unrelated,

$$\text{cov}(\hat{\sigma}^2_{A_p},\hat{\sigma}^2_{A_c}) = \frac{16}{N-1}\text{cov}\left((\overline{P}_{p,sire}-\overline{\overline{P}}_p)^2,(\overline{P}_{c,sire}-\overline{\overline{P}}_c)^2\right),$$

where the covariance now refers to a single sire. Using Equation B2,

$$\text{cov}\left((\overline{P}_{p,sire}-\overline{\overline{P}}_p)^2,(\overline{P}_{c,sire}-\overline{\overline{P}}_c)^2\right) = 2\text{cov}^2(\overline{P}_{p,sire},\overline{P}_{c,sire}) = \frac{2\sigma^2_{A_{pc}}}{16}.$$

Hence,

$$\mathrm{cov}(\hat{\sigma}^2_{A_p}, \hat{\sigma}^2_{A_c}) = \frac{2\sigma^2_{A_{pc}}}{N-1}. \tag{A7}$$

In summary, $SE(\hat{r}_{pc})$ follows from substituting Equations A2, A4, A6 and A7 into Equation A1, and taking the square root. The resulting expression is complex and therefore not given.

The result can be simplified considerably by using the hunch that the effective number of sires is determined by the square of reliability,

$$N_{eff} \approx \rho^4 (N-1), \tag{A8}$$

where ρ^4 is the square of the reliability of the sire EBVs,

$$\rho^2 = \frac{\frac{1}{4}\sigma^2_A}{\mathrm{var}(\bar{P})}. \tag{A9}$$

Substituting Equation A8 into the expression for $SE(\hat{r}_{pc})$ following from Equations A1 through A7 yields Equation 1. Equation A8 derives from the idea that reliability summarizes the quality of the sire EBV as a measure of the true sire breeding value. In our case, interest is in the variance of the variance, so we are on the quadratic scale, which suggests the use of ρ^4. Below is a formal derivation of Equation A8 for a simplified case.

Equation A8: A rigorous proof of Equation A8 for a nested full-half sib scheme is very tedious. We, therefore, only give a proof for a non-nested half-sib design with *N* sires, each with *n* offspring, without full-sib groups within half sib families. The stochastic simulations confirm that the result extends by analogy to the nested full-half sib design with common-litter effects. For a half sib design, the trait model is

$$P = s + e.$$

The ANOVA is given by

$$EMS_{sires} = n\sigma^2_s + \sigma^2_e,$$

$$EMS_{error} = \sigma^2_e.$$

Hence,

$$\hat{\sigma}^2_s = \frac{MS_{sires} - MS_{error}}{n}.$$

Following the derivation used for Equation A4,

$$\mathrm{var}(\hat{\sigma}_s^2) = 2\frac{EMS_{sire}^2 / df_{sire} + EMS_{error}^2 / df_{error}}{n^2}$$

$$= 2\frac{(n^2\sigma_s^4 + 2n\sigma_s^2\sigma_e^2 + \sigma_e^4)/(N-1) + \sigma_e^4/(N(n-1))}{n^2}$$

$$= 2\left[\frac{\sigma_s^4 + 2\sigma_s^2\sigma_e^2/n + \sigma_e^4/n^2}{N-1} + \frac{\sigma_e^4}{Nn^2(n-1)}\right]$$

$$\approx 2\left[\frac{\sigma_s^4 + 2\sigma_s^2\sigma_e^2/n + \sigma_e^4/n^2}{N-1}\right]$$

The last step assumes that, in the third line, the second term is usually much smaller than the first term. (This is reasonable unless n is very small and h^2 is small; Hence, unless ρ^2 is very small). The squared reliability of sire EBVs equals

$$\rho^4 = \frac{\sigma_s^4}{\sigma_s^4 + 2\sigma_s^2\sigma_e^2/n + \sigma_e^4/n^2}$$

Hence,

$$\mathrm{var}(\hat{\sigma}_s^2) \approx \frac{2\sigma_s^4}{(N-1)\rho^4}.$$

Finally, using Equation A5, and substituting $\mathrm{var}(\hat{\sigma}_s^2) = \frac{2\sigma_s^4}{N_{eff}}$ yields Equation A8.

Part B

Though expressions derived here can also be found in multivariate texts, they are included for reasons of completeness. In the following, x and y are bivariate normal variates, μ denotes the mean, σ^2 the variance, σ_{xy} the covariance, ρ the correlation coefficient, b_{yx} the regression coefficient of y on x, and E the expectation.

Var(xy) By the law of total variance,

$$\mathrm{var}(xy) = \mathrm{E}[\mathrm{var}(xy \mid x)] + \mathrm{var}[\mathrm{E}(xy \mid x)].$$

In the first term, $\mathrm{var}(xy \mid x) = x^2(1-\rho^2)\sigma_y^2$, so that

$$\mathrm{E}[\mathrm{var}(xy \mid x)] = (\mu_x^2 + \sigma_x^2)(1-\rho^2)\sigma_y^2.$$

In the second term, $\mathrm{E}(xy\,|\,x)$ = $x[\mu_y + b_{yx}(x-\mu_x)]$. Thus $\mathrm{var}\left[\mathrm{E}(xy\,|\,x)\right]$ = $\mu_y^2\sigma_x^2$ + $b_{yx}^2\,\mathrm{var}[x(x-\mu_x)]$ + $2\mu_y b_{yx}\,\mathrm{cov}[x, x(x-\mu_x)]$. Using the non-central moments of the normal distribution, it follows that $\mathrm{var}[x(x-\mu_x)]$ = $\mu_x^2\sigma_x^2 + 2\sigma_x^4$, and that $\mathrm{cov}[x, x(x-\mu_x)]$ = $\mu_x\sigma_x^2$. Collecting terms,

$$\mathrm{var}\left[\mathrm{E}(xy\,|\,x)\right] = \mu_y^2\sigma_x^2 + 2\sigma_{xy}^2 + \frac{\mu_x^2\sigma_{xy}^2}{\sigma_x^2} + 2\mu_x\mu_y\sigma_{xy}.$$

Summing the first and second term yields

$$\mathrm{var}(xy) = \mu_x^2\sigma_y^2 + \mu_y^2\sigma_x^2 + \sigma_x^2\sigma_y^2 + 2\mu_x\mu_y\sigma_{xy} + \sigma_{xy}^2. \tag{B1}$$

Cov(x²,y²) For μ_x = μ_y = 0,

$$\mathrm{cov}(x^2, y^2) \;=\; \mathrm{E}(x^2y^2) - \mathrm{E}(x^2)\mathrm{E}(y^2) \;=\; \mathrm{E}(x^2y^2) - \sigma_x^2\sigma_y^2.$$

The first term follows from conditioning on *x*, $\mathrm{E}(x^2y^2)$ = $\mathrm{E}[x^2\mathrm{E}(y^2\,|\,x)]$, in which $y\,|\,x = b_{yx}x + e$, with $\mathrm{cov}(b_{yx}x, e) = 0$ and $\mathrm{E}(e) = 0$. Thus $\mathrm{E}(y^2\,|\,x)$ = $b_{yx}^2x^2 + (1-\rho^2)\sigma_y^2$. Taking the expectation over *x*, $\mathrm{E}[x^2\,\mathrm{E}(y^2\,|\,x)]$ = $b_{yx}^2\,\mathrm{E}(x^4)$ + $(1-\rho^2)\sigma_x^2\sigma_y^2$. Substituting $\mathrm{E}(x^4) = 3\sigma_x^4$ yields $\mathrm{E}[x^2\mathrm{E}(y^2\,|\,x)]$ = $2\sigma_{xy}^2 + \sigma_x^2\sigma_y^2$. Finally, subtracting the second term yields

$$\mathrm{cov}(x^2, y^2) \;=\; 2\sigma_{xy}^2. \tag{B2}$$

Cov(x²,xy) : For μ_x = μ_y = 0,

$$\mathrm{cov}(x^2, xy) \;=\; \mathrm{E}(x^3y) - \mathrm{E}(x^2)\mathrm{E}(xy) \;=\; \mathrm{E}(x^3y\;) - \sigma_x^2\sigma_{xy}.$$

The first term follows from conditioning on *x*, $\mathrm{E}(x^3y)$ = $\mathrm{E}[x^3\,\mathrm{E}(y\,|\,x)]$. Since $\mathrm{E}[y\,|\,x] = b_{yx}x$, $\mathrm{E}[x^3\,\mathrm{E}(y\,|\,x)]$ = $b_{yx}\mathrm{E}(x^4)$. Substituting $\mathrm{E}(x^4) = 3\sigma_x^4$ yields $3\sigma_x^2\sigma_{xy}^2$. Finally, subtracting the second term yields

$$\mathrm{cov}(x^2, xy) \;=\; 2\sigma_x^2\sigma_{xy}. \tag{B3}$$
